# The Relationship Between Eating Habits and Anthropometric Values in High School Adolescents

**DOI:** 10.3390/life16040618

**Published:** 2026-04-07

**Authors:** Lucija Firi, Dubravka Mihaljević, Ivan Prošić, Kristina Kralik, Ana Stupin, Ivana Jukić, Ines Drenjančević

**Affiliations:** 1 Health Centre Osijek-Baranja County, 31000 Osijek, Croatia; lucija.firi@gmail.com; 2 Department of Nephrology, Clinical Hospital Centre Osijek, J. Huttlera 4, 31000 Osijek, Croatia; dmihaljevic.os@gmail.com; 3 General Hospital Našice, 31500 Našice, Croatia; ivan.prosic02@gmail.com; 4 Department of Medical Statistics and Medical Informatics, Faculty of Medicine Osijek, University Josip Juraj Strossmayer Osijek, J. Huttlera 4, 31000 Osijek, Croatia; kkralik@mefos.hr; 5 Department of Physiology and Immunology, Faculty of Medicine Osijek, University Josip Juraj Strossmayer Osijek, J. Huttlera 4, 31000 Osijek, Croatia or ana.stupin@mefos.hr (A.S.); ivana.jukic@mefos.hr (I.J.)

**Keywords:** anthropometry, body mass index, dietary habits, waist-hip ratio

## Abstract

**Objectives:** The present study aimed to examine the relationship between dietary habits and anthropometric characteristics of high school adolescents. **Participants and Methods:** This cross-sectional study involved randomly selecting 104 students (34 boys and 70 girls) who were 17–19 years old. To evaluate dietary habits, a validated and standardized EPIC-Norfolk frequency food questionnaire was used, and anthropometric characteristics of the participants were assessed. **Results:** The most often consumed foods during the week were meats and fruits, while the least frequently consumed were fish and vegetables. For all participants, intake below the recommended levels was for alpha-carotene, total carbohydrate sugars, vitamin D, milk, dairy products, nuts, and seeds. Intake of proteins and iron was below the recommended levels in girls. Boys had a lower intake of proteins and fruits compared to girls. No differences were observed in the consumption of macronutrients. The median of all anthropometric values was within the reference values. Boys were slightly older and had significantly higher values of body weight and height, BMI, waist circumference, and waist-to-hip-ratio (WHR) compared to girls. In addition, systolic blood pressure (SBP) was significantly higher in males than in females. Participants with increased arterial blood pressure (ABP) showed significantly higher energy intake from fat, sodium intake, and total fat intake compared to those with normal ABP. Participants with increased WHR had significantly higher energy intake, intake of sodium, and total carbohydrates compared to those with normal WHR. **Conclusions**: This study shows that adolescents’ eating habits (particularly intake of fat and sodium) were associated with anthropometric values, ABP, and WHR, which present risks for cardiometabolic diseases in adulthood.

## 1. Introduction

Adolescence represents a transitional period from childhood to adulthood, characterized by significant biological, cognitive, and psychosocial changes; it typically begins between the ages of 10 and 13, coinciding with the onset of puberty and the development of secondary sexual characteristics, and lasts until adulthood, between 18 and 20 years of age [1]. That is a period of life with particular nutritional and energy needs [2,3]. According to the World Health Organization (WHO), the recommended daily energy intake for adolescents with moderate physical activity ranges between 2300 and 3100 kcal for boys and 2100 and 2400 kcal for girls. However, estimating the energy needs of adolescents is challenging due to variations in growth intensity, developmental stages, and physical activity levels [2]. During adolescence, the adoption of health and nutritional habits has a long-term impact, not only on immediate health status but also on health throughout adulthood [4]. Adolescents often begin to make independent food choices, display irregular eating patterns, and consume meals outside the family setting more frequently [4,5].

Consumption of both macronutrients (i.e., carbohydrates, lipids, and proteins) and micronutrients (vitamins and minerals) daily is essential for optimal growth, development, and functionality [3]. Adolescents have increased protein requirements and can tolerate higher lipid intake, although lipid consumption should not exceed 35% of total energy intake [6]. Carbohydrates should constitute 45–60% of daily energy intake [6]. Certain nutrients, including magnesium, zinc, vitamins A and D, iron, phosphorus, calcium, and fiber, are often not consumed in adequate amounts by adolescents. This insufficiency often stems from inadequate consumption of milk, fruits, vegetables, and grains [2]. The intake of processed foods, such as chips, snacks, frozen ready-made meals, fast food, and sweets, is becoming increasingly prevalent across all age groups, particularly among adolescents. Processed foods are calorie-dense but have low nutritional value. This trend is particularly concerning given that adolescents are in a period of heightened nutritional risk due to more frequent absences from home, a desire for independence, the growing importance of physical appearance, and the increasing influence of media [2,7]. Dietary habits, particularly during childhood and adolescence, play a crucial role in the development of risk factors for obesity and all obesity-related non-communicable chronic diseases [8].

Obesity is a significant public health problem worldwide, including Croatia. In 2019, nearly two-thirds of Croatian adults (64.8%) were overweight [9]. A particular problem presents a growing incidence of obesity among school-age children [10]. It is projected that by 2035, the number of children and adolescents aged 5 to 19 living with obesity in the WHO European Region will reach 28 million, representing a 46.3% increase compared to 2020 [11]. The prevalence of overweight and obese children and adolescents in Croatia has increased to 20% in 2022, and this is similar for boys (21%) and girls (19% of overweight/obese) [10]. Moreover, 35% of children aged 8–9 have been overweight or obese [12,13].

It is now clear that dietary habits have a broad impact on various cardiometabolic risk factors. These include obesity, LDL cholesterol, arterial blood pressure (ABP), glucose–insulin regulation, lipoprotein levels and function, oxidative stress, inflammation, endothelial function, liver health, adipocyte metabolism, cardiac function, metabolic rate, weight regulation mechanisms, visceral fat, and the microbiome. The European Region records the highest number of deaths associated with chronic non-communicable diseases (CNCDs), accounting for 90% of all deaths and 85% of all years of healthy life lost due to disability [11]. CNCDs caused by obesity include cardiovascular diseases, gastrointestinal diseases, metabolic diseases, such as type 2 diabetes, certain types of cancer, sleep apnea, osteoarthritis, and kidney, pulmonary, neurological, and psychiatric conditions [14].

While current dietary guidelines primarily emphasize total calorie intake and obesity, one should focus more on the overall effects of diet on health and on diet quality [15]. For example, high sodium intake leads to endothelial dysfunction and shows a positive correlation with increased ABP, resulting in an increased risk for cardiovascular diseases, such as ischemic heart disease and hypertensive heart disease [16,17]. A diet high in sugar-sweetened beverages, processed foods, higher intake of saturated fatty acids (SFAs) and trans-fat, but a low intake of polyunsaturated and monounsaturated fats as well, adversely affects glucose metabolism and insulin resistance and is associated with an increased risk of diabetes [18,19,20]. A diet high in SFAs is also associated with dyslipidemia and atherosclerosis due to an increase in LDL in serum [20]. Nutrient-sparse foods such as concentrated sugars and refined flour products, low fiber intake, consumption of red meat, and imbalance of omega-3 and omega-6 fats all contribute to excess cancer risk [21]. A high intake of SFAs, a high intake of protein, and low amounts of vegetables, fiber, and fruit increase the incidence of inflammatory bowel diseases, such as Crohn’s disease and ulcerative colitis [12]. Processed foods that are high in SFAs and simple sugars have a higher chance of developing a neurological disease, such as Parkinson’s disease, multiple sclerosis, Alzheimer’s disease, and depression [22]. For example, our previous study demonstrated that apparently healthy young adults (medical students) have unhealthy eating habits, consuming less fruits and fish and more sodas and sweet drinks, which increases their risk of future cardiometabolic diseases [23].

Taken together, and given the limited research in Croatia on dietary habits and the risk of obesity and cardiometabolic diseases in adolescents, the aim of the present study was to evaluate whether their dietary habits and anthropometric measurements are balanced, providing insight into their future risk of developing obesity and cardiometabolic diseases later in life. Additionally, we aimed to evaluate whether there are differences in anthropometric measurements between individuals with unbalanced diets and those with healthier dietary habits. We hypothesize that unbalanced dietary habits negatively impact anthropometric measurement outcomes (body weight, BMI, waist-to-hip ratio, ABP) in adolescents, demonstrating their association with increased risk for future cardiometabolic diseases.

## 2. Materials and Methods

This cross-sectional study was carried out among students in three randomly selected secondary schools in the town of Osijek, Croatia, in May 2024. The study was conducted in two classes per school, including students of both sexes aged 17 to 19 years. A total of 104 participants were included in the study. Participation was voluntary, and all participants signed an informed consent form.

Data on dietary habits were collected using the standardized EPIC-Norfolk Food Frequency Questionnaire (FFQ), translated and validated for Croatia [24]. The questionnaire consists of two parts. The first part includes a list of 130 food items divided into 10 groups: meat and fish, bread and savory biscuits, cereals, potatoes, rice and pasta, dairy products and fats, sweets and snacks, soups, sauces and spreads, beverages, and fruits and vegetables. Each item has a specified portion size, and participants select the frequency of consumption for each item from nine categories (ranging from “never or less than once a month” to “six or more times a day”). The second part of the questionnaire contains additional questions, such as the type and amount of milk consumed, the type of fats used in cooking, the amount of visible fat on meat, and dietary supplements used in the past year. A copy of the questionnaire and instructions for completion and coding are available for download on the official EPIC study website [24]. The questionnaire results were analyzed using the FFQ EPIC Tool for Analysis (FETA), a program for calculating food frequency data. A total of 60 nutrients and food types were input into FETA software version 6 (CAMB/PQ/6/1205) for analysis [24]. The participants’ intake levels of individual micronutrients and macronutrients were compared with dietary reference values (DRVs) as recommended by the European Food Safety Authority (EFSA). This comparison was carried out using the DRV Finder*, a tool that provides access to EFSA’s science-based nutrient reference values for healthy populations [25]. Since DRVs vary by life stage and gender, we categorized participants into two groups, “children and adolescents,” for whom DRVs are defined up to the age of 17, and “adults,” for those aged 18 and above. This process resulted in the creation of tables presenting reference values for individual nutrients, differentiated by gender, for the ages of 17, 18, and 19 [19,26]. An additional survey question addressed the frequency of eating meals in restaurants or bakeries for lunch, breakfast, dinner, or during school breaks, as well as the number of times per week participants eat at a bakery.

For each participant, age and sex were recorded, followed by anthropometric measurements: height, body weight, waist circumference, hip circumference, waist-to-hip ratio, body mass index calculation, ABP measurement, and heart rate (HR) measurement. BMI was assessed using the reference growth curves for children and adolescents [27]. Participants classified as underweight according to BMI were excluded from analyses comparing BMI categories because the number of individuals in this category was very small (*n* = 2) and would not allow meaningful statistical comparisons. ABP was recorded as the arithmetic mean of three consecutive measurements taken on the same arm at the visit to school. Simultaneously, the pulse rate of each participant was also documented. ABP measurements were conducted after a 15 min rest period, with 5 min intervals between measurements, in a seated position using an automated oscillometric ABP monitor (Omron M2, manufactured in 2018, Omron Corporation, Kyoto, Japan). Elevated BP and hypertension were defined according to age-specific thresholds based on percentile values for adolescents [28]. The values differ in the age groups 12 to 17 and 18 to 19. For 17-year-olds, elevated BP is ≥90th to <95th percentile, or ≥120/80 mmHg to <95th percentile, and hypertension is BP equal to or above the 95th percentile. In adolescents 18 to 19 years of age, increased ABP is defined as systolic blood pressure (SBP) ≥ 120 mmHg to <140 mmHg or diastolic blood pressure (DBP) ≥ 80 mmHg to <90 mmHg, and hypertension is defined as ABP ≥ 140/90 [28].

### Statistical Analysis

Categorical data are described using absolute and relative frequencies. Differences in categorical variables were tested using the chi-square test. The normality of the distribution of continuous variables was assessed using the Shapiro–Wilk test. Continuous data are presented as mean with standard deviation (SD) or median with interquartile range (IQR), as appropriate. Differences in continuous variables between two independent groups were tested using the independent samples *t*-test or the Mann–Whitney U test, with Hodges–Lehmann median differences and corresponding 95% confidence intervals (CI) reported. The association between continuous variables was assessed using Pearson’s correlation coefficient (r). Bivariate and multivariate logistic regression analyses were performed to assess factors associated with increased ABP. Given the relatively small number of participants with increased ABP, the number of predictors included in the multivariable model was limited to reduce the risk of overfitting the model. Multicollinearity among predictors was assessed before model fitting using the variance inflation factor (VIF). A stepwise procedure was used as an exploratory approach to identify dietary variables independently associated with increased ABP. All *p* values are two-tailed. Statistical analysis was performed using MedCalc^®^ Statistical Software version 23.4.8 (MedCalc Software Ltd., Ostend, Belgium; https://www.medcalc.org; 2026). The research report was prepared in accordance with the guidelines for reporting research findings in biomedical and health sciences (EquatorNetwork).

## 3. Results

**Anthropometric Measurements.** The study included a total of 104 participants, of whom 34 (32.7%) were boys and 70 (67.3%) were girls. Based on measured body weight and height, body mass index (BMI) values ranged from 16.73 to 29.98 kg/m^2^, encompassing categories from underweight to overweight. The median systolic blood pressure was 122 mmHg (range: 99 to 151 mmHg), and the median diastolic blood pressure was 71 mmHg (range: 51 to 91 mmHg). Statistically significant differences between male and female participants were observed for age, body weight, height, body mass index (BMI), waist circumference, waist-to-hip ratio (WHR), and systolic blood pressure. Male participants were slightly older (*t*-test, *p* = 0.003) and had significantly higher values of body weight and height (both *p* < 0.001), BMI (*p* = 0.004), waist circumference (*p* < 0.001), and WHR (*p* < 0.001) compared with female participants. In addition, systolic blood pressure was significantly higher in males than in females (*p* = 0.001). No significant sex differences were observed for hip circumference, waist-to-height ratio (WHtR), diastolic blood pressure, or HR (Table 1).

Based on BMI, two participants (2.0%) were classified as underweight, 91 (85.0%) as having normal weight, and 11 (13.0%) as overweight or obese. Due to the small number, underweight participants were excluded from further analyses. Increased systolic blood pressure values were observed in nine (8.7%) participants, increased diastolic blood pressure in three (2.9%) participants, and increased HR in seven (6.7%) participants. Participants with overweight/obesity had higher median WHR values (Mann–Whitney U test, *p* < 0.001), higher WHtR, and higher median systolic (*p* = 0.020) and diastolic blood pressure (*p* = 0.015) compared with participants of normal weight. No statistically significant difference in HR was observed between BMI categories (Table 2).

Comparisons between participants with normal and increased ABP showed significant differences in body weight, height, waist circumference, and WHR. Participants with increased ABP had higher mean body weight and height (t-test, both *p* = 0.003), as well as significantly higher waist circumference (*p* = 0.002) and higher WHR (*p* = 0.002) compared with those with normal ABP. No significant differences were observed between ABP categories for age, BMI, hip circumference, WHtR, or HR (Table 3).

**Dietary Habits.** For the whole group, the median number of visits to a bakery or restaurant per week was three (interquartile range [IQR] 2 to 4), with values ranging from 0 to 10 visits per week. The majority of participants, 99 (95.2%), primarily consumed meals prepared at home. Lunch was most commonly eaten at home, by 100 (96.2%) participants, followed by breakfast and dinner in 93 (89.4%) participants. During school breaks, however, 76 (73.1%) participants reported most frequently purchasing snacks from a bakery. The weekly frequency of consumption of specific food groups is presented in Table 4. Fruit and meat were the most frequently consumed food groups, whereas fish was consumed least often (Table 4).

**Milk consumption.** Eight participants (7.7%) reported not consuming milk, while the majority consumed full-fat milk, 73 (70.2%). Regarding quantity, 39 (37.5%) participants reported a usual intake of 1.5 dL of milk.

**Fat use in food preparation.** Vegetable oil was the most frequently used fat for frying, reported by 61 (58.7%) participants, followed by lard, reported by 24 (23.1%) participants, whereas vegetable shortening was used least often. For baking, butter was the most common fat, reported by 60 (57.7%) participants (57.7%), followed by margarine, reported by 34 (32.7%) participants. Lard and vegetable oil were seldom used for baking, and eight (7.7%) participants (7.7%) reported not using any fat for baking.

**Nutrient and food group intake.** The highest prevalence of intake below recommendations was observed for alpha-carotene, total carbohydrate sugars, vitamin D, milk and dairy products, and nuts and seeds. A significantly higher proportion of men than women were below the recommended intake of protein (*p* = 0.018) and fruit (*p* < 0.001), and significantly more women than men had below the recommended intake of iron (*p* = 0.032). Significantly more women than with an established menstrual cycle had not met the recommended daily intake of iron (χ^2^ test, *p* = 0.032), protein (*p* = 0.018), and fruits (*p* < 0.001) (Supplemental Table S1).

**Dietary intake by anthropometric and ABP categories.** No significant differences in energy or macronutrient intake were observed between participants with normal weight and those classified as overweight based on BMI categories. Participants with an elevated waist-to-hip ratio (WHR) had significantly higher energy intake compared to those with a normal WHR (median: 2565.1 vs. 1942.9 kcal; Mann–Whitney U test, *p* = 0.012). Additionally, the elevated WHR group had higher intakes of sodium (*p* = 0.042) and total carbohydrates in grams (*p* = 0.040). No significant differences were observed in the percentage contribution of macronutrients.

When stratified by ABP status, participants with increased ABP showed significantly higher energy intake (*p* = 0.005), sodium intake (*p* = 0.005), and total fat intake in grams (*p* = 0.002) compared with those with normal ABP. The percentage of energy derived from carbohydrates was lower (*p* = 0.006), whereas the percentage of energy from fat was higher (*p* < 0.001) in participants with increased ABP (Table 5).

Pearson’s correlation analysis identified several statistically significant associations between dietary intake variables and both anthropometric and blood pressure measures; however, all observed correlations reflected small effect sizes (|r| < 0.400). A weak negative association was found between the percentage of energy derived from carbohydrates and both WHR (r = −0.267; *p* = 0.006) and systolic blood pressure (r = −0.343; *p* < 0.001). Conversely, the percentage of energy from fat showed weak but significant positive correlations with systolic (r = 0.198; *p* = 0.044) and diastolic blood pressure (r = 0.242; *p* = 0.013). No additional statistically significant correlations were observed (Table 6).

In bivariate logistic regression analysis, female gender was associated with a lower likelihood of having increased ABP (OR = 0.21; *p* = 0.002). Increased WHR was associated with a more than fourfold increase in the likelihood of having high ABP (OR = 4.65; *p* = 0.041). Among dietary variables, higher energy intake (OR = 1.001; *p* = 0.029) and sodium intake (OR = 1.000; *p* = 0.020) were associated with increased ABP; however, the magnitude of these associations was small, which likely reflects the fact that the odds ratios represent the effect per one-unit increase in the respective dietary variables. In addition, higher total fat intake (OR = 1.01; *p* = 0.007) and higher percentage of energy derived from fat (OR = 1.39; *p* = 0.034) were associated with increased likelihood of increased ABP, while higher percentage of energy from carbohydrates was inversely related to the outcome (OR = 0.89; *p* = 0.007).

In the multivariate logistic regression analysis using the stepwise method, the percentage of energy derived from carbohydrates and fat remained independently associated with increased ABP. A higher percentage of energy intake from fat was associated with higher odds of increased ABP (OR = 1.01; *p* = 0.002), whereas a higher percentage of energy derived from carbohydrates was associated with lower odds of the outcome (OR = 0.87; *p* = 0.003). The overall model was significant (χ^2^ = 20.6; *p* < 0.001) and explained between 18% (Cox–Snell R^2^) and 28% (Nagelkerke R^2^) of the variance in increased ABP, with an overall classification accuracy of 81%. Given the relatively small number of participants with increased ABP, these findings should be interpreted with caution (Table 7).

## 4. Discussion

This study aimed to explore the relationship between dietary habits and anthropometric characteristics in adolescents, hypothesizing that an unbalanced diet was associated with increased key anthropometric measures such as body weight, BMI, WHR, ABP, and HR. To achieve this, the validated food frequency questionnaire (EPIC-Norfolk FFQ) was used alongside the anthropometric measurements, taking into account any sex-based differences.

The most important findings of the present study are as follows. (a) The majority of participants had BMI values within the reference range, with a median BMI of 22.22 kg/m^2^. (b) High intake of total fat, high energy intake from fat, and higher sodium intake were significantly associated with increased ABP. (c) Participants with a higher WHR had significantly higher energy, sodium, and total carbohydrate intake. (d) An increased WHR was significantly positively correlated with increased ABP. Altogether, these findings suggest that already in adolescence, unhealthy eating habits are related to increased central obesity and early development of increased ABP, known cardiometabolic risks in adulthood, which is the most salient conclusion of the present study.

BMI is an anthropometric measure frequently used to assess nutritional status. However, a limitation of BMI is its inability to differentiate between fat tissue and other types of tissue. Therefore, the World Health Organization (WHO) recommended additional measurements, such as waist and hip circumference (WHR), as alternatives to BMI for a more accurate estimation of obesity-related comorbidity risk [29]. For children and adolescents, BMI-based obesity assessment involves the use of reference growth curves, which account for age and sex [29]. In the present study, the majority of students had normal BMI; only 11 (out of 104) were overweight/obese, despite a nutritionally unbalanced diet. Furthermore, no association was observed between energy intake, nutrient intake, and food groups with BMI values. Although BMI was not much different from that observed in other studies in Croatian school children [30], the prevalence of overweight/obesity in the present study is lower than previously published in schoolchildren in other parts of Croatia [30]. For example, the prevalence of overweight and obesity in primary school children in 2019, before the lockdown, was 21% in Primorsko-Goranska County, Croatia. During the lockdown, the prevalence rose to 24% [30]. This is in agreement with the observed BMI increases in studies involving children of similar ages in Jordan, Greece, Palestine, and Spain [17,18,19,20]. Our study was performed post-lockdown; thus, a lower overweight/obesity portion of adolescents in our study group may be considered positive, although this is paradoxical to their unhealthy eating habits.

Waist circumference (WC), waist-to-height ratio (WHtR), and waist-to-hip ratio (WHR) are the most common indices used to define abdominal obesity [31]. The present study demonstrated a significant impact of unbalanced diets on WHR. The results indicate that higher WHR values correlated with increased intake of energy from fat and increased sodium intake. This dietary pattern is characteristic of individuals with obesity/overweight and increased WHR, which are associated with an increased risk of metabolic syndrome [32]. Importantly, higher WHR was associated with a more than fourfold increase in the likelihood of increased ABP, as shown by bivariate logistic regression analysis. Indeed, approximately 20% of participants had increased l ABP. Similar findings were reported in a study by Martinis et al., where increased WHR, WC, and WHtR were linked to increased ABP and higher consumption of pastries, white bread cereals, biscuits, snacks, meat, and cured meat products, along with lower fish intake [33]. Similarly, recent secondary analysis of an observational study that included 192 adolescents who were 10–16 years old showed an inverse relationship of food intake quality and anthropometric indices related to visceral obesity, BMI, WC, and insulin resistance [32]. In addition to statistical significance, the magnitude of the observed differences should also be considered. The mean and median differences with corresponding confidence intervals presented in the tables provide an estimate of effect size and indicate that most observed differences were relatively modest. Although significant differences were observed for some variables, the magnitude of these differences was relatively small, indicating that their practical or clinical relevance may be limited.

Higher energy intake and sodium intake, as well as higher total fat intake and higher percentage of energy derived from fat, were statistically significant predictors of increased ABP, while a higher percentage of energy from carbohydrates was inversely related to ABP. Female sex was associated with lower risks of increased ABP. It should also be noted that the observed correlations between dietary variables and anthropometric or ABP measures were relatively weak (|r| < 0.400), suggesting that these associations explain only a small proportion of the variability in the studied outcomes. A study by Martinis et al. revealed that out of 111 boys, 31 (27.93%) had elevated BP, while 35 (14.69%) of 246 girls had elevated BP. It was notable that the proportion of boys with high BP was nearly twice that of girls [33], which is similar to our study. In agreement with Martinis et al., boys were slightly older and had significantly higher values of body weight and height, waist circumference, and WHR compared to girls and significantly higher systolic blood pressure compared to girls. Our recent study has revealed that hypertensive children had significantly higher estimated daily salt intake, which was positively associated with ABP and BMI. Furthermore, higher BMI and higher metabolic rate (kcal) (such as in hypertensive children) were positively associated with ABP [34]. The prevalence of increased ABP in Central European studies among adolescents ranges from 2.2% in Switzerland to 4.9% in Poland [33,35,36]. Data from Southern Europe indicate a higher prevalence, with hypertension rates among adolescents estimated to be at least 9% in Turkey and up to 13% in Portugal [28]. In a study by Jureša et al., the prevalence of high-normal and elevated BP was found in 965 healthy students (48.7% girls) from the final grade of primary school (8th grade) and the 3rd year of high school in Croatia. However, data to accurately assess the prevalence of elevated BP among adolescents in Croatia are still insufficient [33,35]. There are several possible mechanisms triggered by high sodium intake that may contribute to increase in arterial blood pressure, including alterations in renal sodium handling, increased intravascular volume, and activation of the renin–angiotensin–aldosterone system, particularly in the presence of obesity [37].

Higher consumption of fresh fruits, non-starchy vegetables, and dairy in early adolescence has been shown to decrease the accumulation of cardiovascular risks in late adolescence, as demonstrated in the NHLBI Growth and Health Study on 1369 girls [38]. The large-scale study by the WHO’s European Childhood Obesity Surveillance Initiative (COSI) highlights fruit and vegetable consumption as an area for improvement in adolescent diets [26]. The COSI study found that 42.5% of children consume fruit daily, although the results vary significantly between countries [39]. For Croatia, the COSI study indicated that 33.8% of children eat fruit daily, while 29.1% consume fruit most days of the week (4–6 times) [39]. The same study revealed that only 22.6% of children consume vegetables every day, and the largest proportion (41.3%) eats vegetables only some days of the week (1–3 times). Our present study yielded favorable results, showing that the median fruit consumption among adolescents is five times per week, making it the most frequently consumed food group weekly. The median vegetable consumption was found to be four times per week, which is more than in previously published studies. We could only speculate on the reason for this difference. One possible explanation may be consumption of the homemade meals, which have better nutritional quality compared to take-out or fast-food or bakery goods [40,41], nutritional literacy, which usually goes with higher socioeconomic backgrounds (such as our studied population had), and healthier food options related to specific initiatives promoting healthier eating among adolescents (e.g., free fruits in school during school breaks). In addition to fruit, meat is the next most commonly consumed food group, with a median frequency of five times per week. Chicken meat is mostly consumed, with a median of four times per week. These results align with a study by Martinis et al., [42], which demonstrated that in Eastern Croatia, white meat is consumed much more frequently (72.3%) than red meat [2]. The present study found that participants consume fish very rarely, with a median frequency of only once per week. In Europe, dietary recommendations suggest consuming between 100 g and 482 g per week, equivalent to one or two servings [43]. The low frequency of fish consumption aligns with the findings of Martinis et al., which reported that the majority of participants from Eastern Croatia (65.9%) stated they never or rarely eat fish (at least once a week). In contrast, the same study showed that in Dalmatia, 58.1% of participants frequently or always consumed fish at least once per week [3]. Since 2018, a declining trend in fish consumption has been observed across the European Union, and the results vary greatly in different countries. In Spain, the average weekly per capita fish consumption is 240 g, compared to 110 g in Italy and just 60 g in France, which reports the lowest levels [44]. Fish consumption varies not only between countries but also among individuals. Data from the National Health and Nutrition Examination Surveys indicate that younger individuals, as well as those with lower income and education levels, tend to consume the least fish. Similarly, Polish studies reveal that 14.1% of adolescents consume fish less than once a week, while 26.2% do not consume fish at all. Regarding frying fats, 61 participants (58.7%) predominantly use vegetable oil, 24 (23.1%) use lard, and the least used is plant-based solid fats, which is supported by Martinis et al.’s study. They showed that most participants from Eastern Croatia (55.8%) frequently or always use other vegetable oils instead of olive oil [3].

The results of this study revealed that a significant number of girls, compared to boys, do not consume adequate amounts of iron, protein, or fruit. In contrast, a study analyzing the dietary habits of adolescents in Slovenia found that the female population there had insufficient intake of vitamin D, folates, and calcium [45], which was not observed in our study. Insufficient calcium intake is often associated with low consumption of milk and dairy products; however, the present study found that only eight participants did not consume milk, while more than one-third consumed at least 1.5 dL per day. Iron deficiency is a major global public health concern, with estimates indicating that 47% of preschool children and 25% of school-aged children worldwide do not consume adequate amounts of iron [46]. A study on Slovenian adolescents showed no issues with iron intake [47], with half of the female participants meeting the recommended dietary allowance, though iron intake tended to decrease with age [43]. This contrasts with the findings of this study, which highlighted insufficient iron intake in a significant portion of the female population.

### Limitations and Recommendations

One limitation of the study was not assessing the physical activity of adolescents. However, since the aim was to correlate nutritional intake to anthropometric parameters, the study protocol was focused on dietary assessment in order to reduce methodological complexity and avoid additional sources of measurement bias. Interestingly, there was no difference in total energy intake or macronutrient intake among participants with normal weight and those classified as overweight based on BMI categories in the present study. Therefore, future studies should address physical activity to better assess the recommended energy intake for each individual and provide a more comprehensive understanding of adolescents’ lifestyle habits. The study population exhibited a relatively homogeneous distribution of body mass index (BMI), with most adolescents within the normal range and only a small proportion classified as overweight or obese. This limited variability may have reduced the ability to detect associations with BMI. Additionally, a few adolescents presented with increased ABP, particularly diastolic blood pressure, which may have limited the statistical power for analyses related to ABP outcomes. The study involved multiple statistical comparisons across several anthropometric and dietary variables, so the risk of type I error cannot be excluded. Some significant findings may, therefore, reflect random variation rather than true associations. As the analyses were primarily exploratory and hypothesis-generating, no formal correction for multiple testing was applied. Consequently, these results should be interpreted with caution and validated in larger studies.

## 5. Conclusions

The results of the present study show that the dietary habits of adolescents are related to anthropometric measures. While dietary habits were not significantly associated with BMI, they were associated with WHR. The majority of adolescents had BMI values within the reference range, indicating that BMI is not a relevant indicator of diet quality, while WHR (abdominal fat) is. Importantly, increased WHR seems to be an important risk factor for future hypertension since it was associated with a fourfold increase in the likelihood of having increased ABP.

Increased intake of sodium, energy from fat, and total fat intake was associated with increased systolic blood pressure, thus presenting potential risk for future development of arterial hypertension and other related cardiovascular diseases. However, these associations were weak. Altogether, the results of this study may present a basis for the creation of wider studies, in different regions and planning of public health actions directed towards school children, parents, and teachers, to increase their awareness of risks related to unhealthy dietary habits.

## Figures and Tables

**Table 1:** Anthropometric characteristics, ABP, and HR of participants stratified by sex.

	Mean (SD)			Mean Difference (95% CI)	*p* Value
	All Participants	Male (*n* = 34)	Female (*n* = 70)		
Age (years)	17.8 (0.6)	18 (0.6)	17 (0.5)	−0.4 (−0.6 to −0.1)	**0.003**
Weight (kg)	67 (12.9)	80 (12.8)	62 (7.7)	−18 (−22.9 to 13.3)	**<0.001**
Height (cm)	174 (9.7)	184 (6.8)	169 (5.9)	−16 (−18.4 to 13.3)	**<0.001**
BMI (kg/m^2^)	22.22 (2.8)	23.31 (3.2)	21.68 (2.4)	−1.6 (−2.7 to −0.5)	**0.004**
Waist circumference (cm)	78.0 (10.1)	85.3 (8.8)	74.5 (8.7)	−10.8 (−14.4 to −7.2)	**<0.001**
Hip circumference (cm)	99.9 (10.3)	102.6 (8.9)	98.6 (10.8)	−4 (−8.2 to 0.2)	0.061
WHR	0.78 (0.1)	0.83 (0.1)	0.76 (0.1)	−0.07 (−0.1 to −0.05)	**<0.001**
WHtR	0.45 (0.05)	0.46 (0.04)	0.44 (0.05)	−0.20 (−0.04 to 0.001)	0.059
ABP (mmHg)					
Systolic (SBP)	122 (11.5)	128 (11.4)	119 (10.3)	−8.4 (−13.3 to −4.4)	**0.001**
Diastolic (DBP)	69 (8.4)	70 (9.0)	69 (8.0)	−1.2 (−4.7 to 2.3)	0.491
HR	80 (12.4)	78 (11.5)	81 (12.7)	3.5 (−1.7 to 8.6)	0.183

Values are presented as mean (standard deviation). Differences represent mean differences with 95% confidence intervals (CIs). Comparisons between groups were performed using the independent samples *t*-test. Abbreviations: SD—standard deviation; CI—confidence interval; BMI—body mass index; WHR—waist-to-hip ratio; WHtR—waist-to-height ratio; ABP—arterial blood pressure; HR—heart rate. **Bold values** denote statistical significance (*p* < 0.05).

**Table 2:** Differences in WHR, ABP, and HR across BMI categories.

	Median (IQR)			Median Differences (95% CI)	*p* Value
	All Participants	Normal Weight (*n* = 91)	Overweight/ Obese (*n* = 11)		
WHR	0.78 (0.74–0.83)	0.77 (0.73–0.81)	0.86 (0.84–0.91)	0.10 (0.06 to 0.13)	**<0.001**
WHtR	0.44 (0.41–0.47)	0.44 (0.41–0.47)	0.52 (0.51–0.57)	0.09 (0.06 to 0.12)	**<0.001**
ABP (mmHg)					
Systolic (SBP)	123 (114–129)	120 (112–128)	128 (123–144)	11 (1.7 to 20.0)	**0.020**
Diastolic (DBP)	69 (63–76)	67 (63–74)	76 (69–82)	8 (1.3 to 14.0)	**0.015**
HR	80 (72–88)	80 (72–88)	80 (73–90)	1 (−7 to 8.0)	0.623

Values are presented as median (IQR). Differences represent Hodges–Lehmann median differences with 95% confidence intervals (CIs). Comparisons between groups were performed using the Mann–Whitney U test. Two underweight participants were excluded due to small numbers. Abbreviations: IQR—interquartile range; CI—confidence interval; WHR—waist-to-hip ratio; WHtR—waist-to-height ratio; ABP—arterial blood pressure; HR—heart rate. **Bold values** denote statistical significance (*p* < 0.05).

**Table 3:** Anthropometric characteristics and body composition parameters according to the ABP category.

	Mean (SD)			Mean Difference (95% CI)	*p* Value
	All Participants	Normal ABP (*n* = 83)	Increased ABP (*n* = 21)		
Age (years)	17.8 (0.6)	17.8 (0.7)	17.9 (0.4)	0.2 (−0.1 to 0.5)	0.224
Weight (kg)	67 (12.9)	66 (11.2)	75 (16.3)	9.4 (3.4 to 15.3)	**0.003**
Height (cm)	174 (9.7)	170 (9.2)	179 (9.7)	7.1 (2.6 to 11.6)	**0.003**
BMI (kg/m^2^)	22.22 (2.8)	21.98 (2.6)	23.13 (3.3)	1.2 (−0.2 to 2.5)	0.090
Waist circumference (cm)	78.02 (10.1)	76.49 (8.9)	84.07 (12.1)	7.6 (2.9 to 12.3)	**0.002**
Hip circumference (cm)	99.92 (10.3)	99.3 (10.2)	102.5 (10.8)	3.2 (−1.8 to 8.2)	0.206
WHR	0.78 (0.1)	0.77 (0.06)	0.82 (0.05)	0.04 (0.02 to 0.07)	**0.002**
WHtR	0.45 (0.05)	0.44 (0.05)	0.47 (0.06)	0.02 (−0.001 to 0.05)	0.064
ABP (mmHg)					
Systolic (SBP)	122 (11.5)	118 (7.9)	139 (6.2)	21.3 (17.6 to 24.9)	**<0.001**
Diastolic (DBP)	69 (8.4)	67 (6.9)	78 (8.4)	10.7 (7.2 to 14.2)	**<0.001**
HR	80 (12.4)	81 (12.3)	77 (12.9)	−3.4 (−9.4 to 2.6)	0.261

Values are presented as mean (standard deviation). Differences represent mean differences with 95% confidence intervals (CIs). Comparisons between groups were performed using the independent samples *t*-test. Abbreviations: SD—standard deviation; CI—confidence interval; BMI—body mass index; WHR—waist-to-hip ratio; WHtR—waist-to-height ratio; ABP—arterial blood pressure; HR—heart rate. **Bold values** denote statistical significance (*p* < 0.05).

**Table 4:** Weekly frequency of consumption of selected food groups.

	Median (Interquartile Range)	Minimum– Maximum
Vegetables	4 (3–6)	0–21
Salat	4 (2–5)	1–7
Fruit	5 (3–6)	1–10
Fish	1 (1–2)	1–6
Meat	5 (3–6)	1–12

**Table 5:** Dietary intake according to BMI, WHR, and ABP categories.

	Median (IQR)		Median Differences (95% CI)	*p* Value
BMI	Normal Weight (*n* = 91)	Overweight/ Obese (*n* = 11)		
Total carbohydrates	216.4 (149.6–291.4)	226.9 (186.4–287.3)	11.9 (−63.2 to 74.2)	0.710
Percentage of energy from carbohydrates	44 (41–49)	42 (36–51)	−3 (−9 to 5)	0.468
Energy (kcal)	1958.9 (1455.8–2592.9)	2415.9 (1715.9–2950.3)	288.2 (−317.1 to 925.6)	0.404
Sodium	2973.2 (2054.0–4314.8)	2920.1 (2691.4–3803.1)	139.0 (−915.7 to 1109.0)	0.768
Total sugars	100.2 (69.5–151.3)	114.8 (76.9–158.2)	4.3 (−38.7 to 45.6)	0.878
Percentage of energy from sugars	21 (18–26)	18 (15–27)	−3 (−7 to 3)	0.294
Fat	78.2 (61.4–116.9)	96.8 (72.2–122.6)	10.6 (−20.2 to 40.3)	0.488
Percentage of energy from fat	17 (16–18)	17 (15–19)	−1 (−2 to 1)	0.506
**WHR**	**Normal (** ***n*** **= 96)**	**Elevated (** ***n*** **= 8)**	**Median Differences** **(95% CI)**	***p*** * Value
Total carbohydrates	211.7 (149.9–280.2)	334.9 (255.8–383.1)	103.9 (7.8 to 168.9)	**0.043**
Percentage of energy from carbohydrates	44 (40–48)	45 (38–52)	0 (−6 to 7)	0.961
Energy (kcal)	1942.9 (1445.8–2553.6)	2565.1 (2335.7–3353.7)	773.3 (212.3 to 1298.6)	**0.012**
Sodium	2928.5 (2098.14–3994.7)	4123.6 (3727.3–5599.8)	1316.2 (5.3 to 2368.7)	**0.042**
Total sugars	95.4 (69.7–136.7)	149.2 (131.9–205.4)	54.2 (3.6 to 91.2)	**0.040**
Percentage of energy from sugars	21 (18–26)	23 (16–28.5)	1 (−58 to 8)	0.850
Fat	79.3 (61.5–113.3)	112.6 (78.0–156.4)	23.9 (−12.5 to 63.1)	0.196
Percentage of energy from fat	17 (16–18)	17 (12.5–19)	−1 (−4 to 1)	0.488
**WHtR**	**Normal (≤0.5) (** ***n*** **= 87)**	**Elevated (** ***n*** **= 17)**	**Median Differences** **(95% CI)**	***p*** * Value
Total carbohydrates	216.9 (150.6–281.9)	215.9 (155.7–359.0)	8.1 (−44.4 to 68.4)	0.773
Percentage of energy from carbohydrates	44 (41–49)	41 (37–47)	−4 (−7 to 1)	0.081
Energy (kcal)	1951.01 (1455.8–2575.9)	2328.7 (1662.1–3344.3)	278.7 (−179.74 to 760.7)	0.240
Sodium	2929.3 (2054.0–4147.6)	3490.2 (2540.8–4273.2)	404.6 (−410.5–1128.8)	0.319
Total sugars	100.2 (70.1–139.8)	108.8 (67.6–160.4)	0.86 (−27.2 to 34.6)	0.982
Percentage of energy from sugars	21 (19–26)	16 (16–25)	−3 (−6 to 0)	0.064
Fat	80.4 (62–113.8)	88.3 (62.1–137.1)	5.03 (−15.1 to 29.2)	0.591
Percentage of energy from fat	17 (16–18)	17 (16–19)	0 (−1 to 1)	0.683
**ABP**	**Normal (** ***n*** **= 83)**	**Elevated (** ***n*** **= 21)**	**Median Differences** **(95% CI)**	***p*** * Value
Total carbohydrates	208.6 (147.2–281.9)	246.5 (205.4–299.5)	31.3 (−14.5 to 79.0)	0.183
Percentage of energy from carbohydrates	44 (41–50)	39 (37–45)	−4 (−8 to −2)	**0.006**
Energy (kcal)	1889.9 (1391.7–2442.6)	2527.9 (1980.1–2758.3)	563.7 (189.6 to 935.2)	**0.005**
Sodium	2851.4 (2041.6–3759.5)	4150.4 (2728.3–6030.4)	1151.5 (382.2 to 2033.1)	**0.005**
Total sugars	95.9 (68.7–143.3)	125.8 (87.3–152.2)	17.4 (−9.6 to 42.7)	0.202
Percentage of energy from sugars	21 (18–26)	19 (16–23)	−2 (−5 to 0)	0.091
Fat	76.9 (59.6–103.1)	114.3 (84.9–142.5)	32.8 (12.2 to 54.6)	**0.002**
Percentage of energy from fat	17 (16–18)	18 (18–20)	2 (1 to 2)	**<0.001**

Values are presented as median (IQR). Differences represent Hodges–Lehmann median differences with 95% confidence intervals (CIs). Comparisons between groups were performed using the Mann–Whitney U test. Two underweight participants were excluded due to small numbers. Abbreviations: IQR—interquartile range; CI—confidence interval; BMI—body mass index; WHR—waist-to-hip ratio; WHtR—waist-to-height ratio; ABP—arterial blood pressure. * **Bold values** denote statistical significance (*p* < 0.05).

**Table 6:** Pearson’s correlation between dietary intake variables and BMI, WHR, and ABP.

	Pearson’s Correlation Coefficient R (*p* Value)				
	BMI	WHR	WHtR	SBP	DBP
Total carbohydrates	0.024 (0.807)	0.051 (0.604)	−0.059 (0.551)	−0.009 (0.929)	0.029 (0.769)
Percentage of energy from carbohydrates	−0.143 (0.147)	**−0.267 (0.006)**	−0.163 (0.092)	**−0.343 (<0.001)**	−0.133 (0.177)
Energy (kcal)	0.092 (0.354)	0.166 (0.092)	−0.003 (0.981)	0.115 (0.244)	0.080 (0.419)
Sodium	−0.100 (0.314)	0.047 (0.636)	−0.053 (0.59)	0.166 (0.091)	**0.255 (0.009)**
Total sugars	0.028 (0.779)	0.048 (0.636)	−0.042 (0.668)	−0.038 (0.700)	−0.018 (0.855)
Percentage of energy from sugars	−0.073 (0.460)	−0.151 (0.127)	−0.110 (0.270)	**−0.260 (0.008)**	−0.153 (0.121)
Fat	−0.012 (0.900)	0.135 (0.171)	−0.048 (0.626)	**0.199 (0.043)**	0.195 (0.048)
Percentage of energy from fat	−0.162 (0.101)	0.005 (0.960)	−0.085 (0.385)	**0.198 (0.044)**	**0.242 (0.013)**

Values represent Pearson correlation coefficients (r) with corresponding *p* values. Abbreviations: BMI—body mass index; WHR—waist-to-hip ratio; WHtR—waist-to-height ratio; SBP—systolic blood pressure; DBP—diastolic blood pressure; ABP—arterial blood pressure. **Bold values** denote statistical significance (*p* < 0.05).

**Table 7:** Bivariate and multivariate logistic regression analyses for increased ABP.

	Bivariate Logistic Regression			Multivariate Logistic Regression (Stepwise Method)		
	β	*p* Value	OR (95% CI)	β	*p* Value	OR (95% CI)
Sex (F)	−1.57	**0.002**	0.21 (0.08 to 0.57)			
Age	0.49	0.219	1.64 (0.74 to 3.66)			
WHR (elevated)	1.54	**0.041**	4.65 (1.06 to 20.4)			
WHtR	8.01	0.071	30.5 (0.48 to 1847.3)			
Total carbohydrates	0.003	0.224	1.002 (0.99 to 1.01)			
Percentage of energy from carbohydrates	−0.11	**0.007**	0.89 (0.83 to 0.97)	−0.13	**0.003**	0.87 (0.80 to 0.96)
Energy (kcal)	0.001	**0.029**	1.001 (1.000 to 1.001)			
Sodium	0.000	**0.020**	1.000 (1.000 to 1.001)			
Total sugars	0.003	0.267	1.003 (0.99 to 1.01)			
Percentage of energy from sugars	−0.07	0.088	0.93 (0.85 to 1.01)			
Fat	0.01	**0.007**	1.01 (1.001 to 1.02)	2.85	**0.002**	1.01 (1.01 to 1.02)
Percentage of energy from fat	0.33	**0.034**	1.39 (1.03 to 1.88)			

Abbreviations: β—regression coefficient; OR—odds ratio; CI—confidence interval; WHR—waist-to-hip ratio; WHtR—waist-to-height ratio; ABP—arterial blood pressure. Variables significant in bivariate analyses were considered for inclusion in the multivariate logistic regression model. To avoid model overfitting, given the limited number of outcome events, only selected predictors were retained in the final model. **Bold values** denote statistical significance (*p* < 0.05).

## Data Availability

The original contributions presented in this study are included in the article/Supplementary Materials. Further inquiries can be directed to the corresponding author.

## Supplementary Materials

The following supporting information can be downloaded at: https://www.mdpi.com/article/10.3390/life16040618/s1, Table S1: Distribution of participants not meeting recommended energy, nutrient, and food group intake by sex.

## Abbreviations

The following abbreviations are used in this manuscript:

ABP	arterial blood pressure
BMI	body mass index
CNCDs	chronic non-communicable diseases
COSI	Childhood Obesity Surveillance Initiative
DBP	diastolic blood pressure
EFSA	European Food Safety Authority
FFQ	Food Frequency Questionnaire
HR	heart rate
IQR	interquartile range
LDLs	low-density lipoproteins
SBP	systolic blood pressure
WC	waist circumference
WHO	World Health Organization
WHR	waist-to-hip ratio
WHtR	waist-to-height ratio
