# Supplementary material for: The Relationship Between Eating Habits and Anthropometric Values in High School Adolescents
Source: Life (Basel). 2026 Apr 7;16(4):618. doi: 10.3390/life16040618 (PMC13117922; doi:10.3390/life16040618)
Supplement: Supplementary file 1 [file life-16-00618-s001.zip › life-4201668-supplementary.pdf]

Supplement

Table S1. Distribution of participants not meeting recommended energy, nutrient, and food group intake by sex

Not included as recommended...	Number of participant (%)			P* value
	Male (n = 34)	Female (n = 70)	Total (n = 104)	
Alpha-carotene	34 (100.0)	70 (100.0)	104 (100.0)	-
Beta-carotene	26 (76.5)	42 (60.0)	68 (65.4)	0.09
Calcium	23 (67.6)	48 (68.6)	71 (68.3)	0.92
Total Carbohydrates	2 (5.9)	4 (5.7)	6 (5.8)	0.97
Copper	48 (49.5)	3 (42.9)	51 (49.0)	0.33
Englyst Fiber – Non-starch Polysaccharides (NSP)	29 (85.3)	49 (70.0)	78 (75.0)	0.09
Iron	17 (50.0)	50 (71.4)	67 (64.4)	**0.03**
Total folates	25 (73.5)	52 (74.3)	77 (74.0)	0.93
Iodine	12 (35.3)	38 (54.3)	50 (48.1)	0.07
Potassium	18 (52.9)	41 (58.6)	59 (56.7)	0.59
Energy	23 (67.6)	57 (81.4)	80 (76.9)	0.12
Magnesium	14 (41.2)	29 (42.6)	43 (42.2)	0.89
Manganese	26 (76.5)	47 (67.1)	73 (70.2)	0.33
Sodium	28 (82.4)	54 (77.1)	82 (78.8)	0.54
Niacin	2 (5.9)	6 (8.6)	8 (7.7)	0.63
Proteins	8 (23.5)	5 (7.1)	13 (12.5)	**0.02**
Vitamin A (Retinol)	14 (41.2)	42 (60.0)	56 (53.8)	0.07
Vitamin B2 (Riboflavin)	11 (32.4)	30 (42.9)	41 (39.4)	0.30
Selenium	15 (44.1)	42 (60.0)	57 (54.8)	0.13
Vitamin B1-Thiamine	3 (8.8)	6 (8.6)	9 (8.7)	0.97
Total carbohydrates-Sugars	32 (94.1)	66 (94.3)	98 (94.2)	0.97
Vitamin B12 (Cobalamin)	4 (11.8)	20 (28.6)	24 (23.1)	0.06
Vitamin B6 (Pyridoxine)	10 (29.4)	23 (32.9)	33 (31.7)	0.72
Vitamin C (Ascorbic acid)	18 (52.9)	30 (42.9)	48 (46.2)	0.33
Vitamin D (Ergocalciferol)	33 (97.1)	68 (97.1)	101 (97.1)	0.98
Vitamin E (Alpha-tocopherol equivalents)	14 (41.2)	30 (42.9)	44 (42.9)	0.87
Zinc	24 (70.6)	50 (71.4)	74 (71.2)	0.93
Fruits	31 (91.2)	44 (62.9)	75 (72.1)	**<0.001**
Meat and Meat Products	25 (73.5)	38 (54.3)	63 (60.6)	0.06
Milk and Dairy Products	29 (85.3)	65 (92.9)	94 (90.4)	0.22
Nuts and seeds	29 (85.3)	67 (95.7)	96 (92.3)	0.06
Vegetables	28 (82.4)	51 (72.9)	79 (76.0)	0.29

Bold values denote statistical significance. *Chi-squared test (Male vs. Female)
